# Dietary Compliance and Quality of Life in Celiac Disease: A Long-Term Follow-Up of Primary School Screening-Detected Patients

**DOI:** 10.3389/fped.2021.787938

**Published:** 2021-12-21

**Authors:** Donatella Iorfida, Francesco Valitutti, Annarita Vestri, Arianna Di Rocco, Salvatore Cucchiara, Riccardo Lubrano, Monica Montuori

**Affiliations:** ^1^Pediatric Gastroenterology and Liver Unit, Department of Maternal and Child Health, Sapienza–University of Rome, Rome, Italy; ^2^EBRIS (European Biomedical Research Institute of Salerno), Salerno, Italy; ^3^Department of Public Health and Infectious Disease, Sapienza–University of Rome, Rome, Italy; ^4^Pediatrics and Neonatology Unit, Department of Maternal and Child Health, Santa Maria Goretti Hospital, Sapienza–University of Rome, Latina, Italy

**Keywords:** quality of life, dietary compliance, follow up, screening, case finding, gluten free diet (GFD), celiac disease, adolescents/young adults

## Abstract

**Background:** Whether the diagnostic approach for celiac disease (CD) can really affect quality of life (QoL) and dietary compliance remains controversial.

**Aims:** This study aimed to evaluate QoL and compliance to gluten-free diet (GFD) in adolescents/young adults diagnosed with CD through a screening strategy during childhood compared to age-matched CD patients diagnosed by case-finding and to assess whether follow-up at a referral center for CD influences compliance and QoL.

**Materials and Methods:** Thirty-seven CD patients who were diagnosed by screening programs (SC-group) and 38 age-matched CD patients diagnosed due to symptoms (CF-group) were enrolled. Patients were asked to answer a questionnaire on QoL, dietary compliance, and follow-up care for CD.

**Results:** Twenty-nine patients of the SC-group (median age 18.0 years, interquartile range [IQR] 16.0–19.0) and 31 patients of the CF-group (median age 17.0 years, IQR 15.5–18.0) completed the questionnaire. No significant difference relating adherence to the GFD and QoL was shown between the two groups. The majority (93.5%) of CF-group regularly had annual follow-up at a referral center compared to 37.9% of the SC-group (*p* < 0.001).

**Conclusion:** The diagnostic strategy does not seem to impact QoL and dietary compliance. However, implementation of follow-up might still be necessary for patients identified through screening.

## Introduction

The number of celiac disease (CD) patients has increased worldwide during the past few decades (1–3). Nevertheless, due to its highly variable clinical presentation, diagnosis is still challenging, and there are many undiagnosed or late-diagnosed cases (4). In some regions, diagnosis took up to 10 years or more, delaying treatment. The time interval between first symptoms and final CD diagnosis in children in five Central European countries was found to be slightly shorter compared with few other small pediatric studies and significantly shorter than reported in adult studies. Nevertheless, an important proportion of children remains undiagnosed for more than 3 years, hence an unacceptable delay (5).

CD largely meets the criteria for a population screening as defined by the World Health Organization (6); unfortunately, this topic is still debated due to some ethical aspects. Firstly, the natural history of the disease is uncertain, since the risks of severe complications in asymptomatic patients, not on a gluten-free diet (GFD), are not entirely clear. Moreover, imposing a permanent and sometimes demanding treatment such as the GFD in patients could impact quality of life (QoL) and dietary compliance. This could even be more crucial in adolescents, who are at risk of follow-up loss prior to and during transition to adult care (7).

To date, whether the diagnostic approach (screening vs case-finding) can really affect adherence to the GFD and QoL remains controversial. Given this background, the primary aim of our study was to investigate QoL and compliance to GFD in adolescents/young adults diagnosed with CD through a screening strategy during childhood compared to age-matched patients diagnosed with CD by case-finding, due to disease-related symptoms. A secondary aim was to assess whether follow-up at a referral center for CD influences dietary compliance and QoL in this age group.

## Materials and Methods

This observational retrospective cohort study was conducted between April and October 2019 at the Pediatric Gastroenterology and Liver Unit of Policlinico Umberto I, Sapienza, University of Rome, Italy.

### Patient Selection and Study Design

Patients included in the study were as follows:

- CD patients (14–20 years old) diagnosed by screening programs carried out in primary schools of the Lazio region (Italy) in 2007 and 2010 (8, 9), which allowed to identification of altogether 45 new CD patients (namely, the screen-detected group or SC-group); and
- CD patients (14–20 years old) diagnosed by age 4 or older because of clinical suspicion (namely, the case-finding group or CF-group).

Major psychiatric disorders or history of cancer were considered exclusion criteria.

A questionnaire was administered to enrolled patients in order to evaluate the following:

- CD-related QoL;
- compliance to GFD; and
- follow-up for CD.

CD patient association membership of either the patient or a first-degree family member was also investigated.

Patients were invited to participate in the study by a physician as part of follow-up consultation or by telephone. Questionnaires were sent to patients via a dedicated email address or were proposed during the outpatient follow-up consultation but then completed in a separate, quiet, non-clinical environment.

### QoL Assessment: The Italian Version of the Celiac Disease-Specific Quality of Life Scale (CD-QoL Scale)

The Italian version of the CD-QoL scale (10) was used for QoL assessment. The CD-QoL scale, developed by Dorn et al. (11), is specific for CD, i.e., designed to specifically assess the profile of CD patients. Higher scores correspond to a better QoL and a lower impact of the disease in daily life (11).

The Italian version of the CD-QoL scale, validated in the study by Zingone et al. has been demonstrated to be a valid tool for QoL assessment in Italian CD patients, not influenced by demographic variables, symptoms at the time of diagnosis, and time from diagnosis (10).

### Assessment of Compliance to the GFD

The Biagi score (12) was applied in order to define compliance, identifying three categories of patients: (1) scores 0 and I identify patients who do not follow the diet adequately; (2) a score of II indicates that the patient makes dietary errors that require correction; and (3) scores III and IV identify patients with strict adherence to the GFD. For statistical analysis, the scores were assessed both individually and in association with the three aforementioned categories.

Validated on an adult population, the Biagi score was also used in two recent pediatric studies (13, 14). In our study, the questionnaire scores were obscured in order not to condition patients' responses.

### Evaluation of Follow-Up

For the definition of follow-up, the two groups were compared on the basis of four modalities: (1) annual follow-up at a referral center for CD; (2) follow-up at a CD referral center, with last check-up dating back more than 3 years; (3) follow-up for CD in primary care settings; and (4) no clinical or serological follow-up for CD. For statistical analysis, the first two modalities were analyzed individually (four-modality follow-up analysis) and also into a single modality corresponding to “follow-up at a referral center for CD” (three-modality follow-up analysis).

### Ethical Aspects

All participants were informed about the aim of the study, and a written consent was obtained for each enrolled patient. Written consent in young adults was provided by patients themselves; in case of patients under 18, a parental written consent and an age-adapted written consent were obtained; different information leaflets about the study were available in plain language and suitable for different age groups.

The study was launched after approval by the Ethical Committee of “Sapienza” University of Rome, Italy (March 2019).

### Statistical Analysis

We could not formally calculate a sample size when the subjects voluntarily participated to the screening, so it is configured as a convenience sample. Continuous variables are presented as mean ± SD or median and IQR, depending on the shape of the distribution curve. Categorical variables are summarized with counts and percentages. Differences between groups (SC-group and CF-group) were evaluated with the independent-samples *t*-test, the Mann–Whitney *U*-test, or chi-square test when appropriate. A *p* < 0.05 was considered statistically significant. We did not use any method of imputation of missing data as we only had one (in the SC-group, CD patient association membership). The open-source software R v. 3.6.3 was used for statistical analysis.

## Results

**Figure 1:**
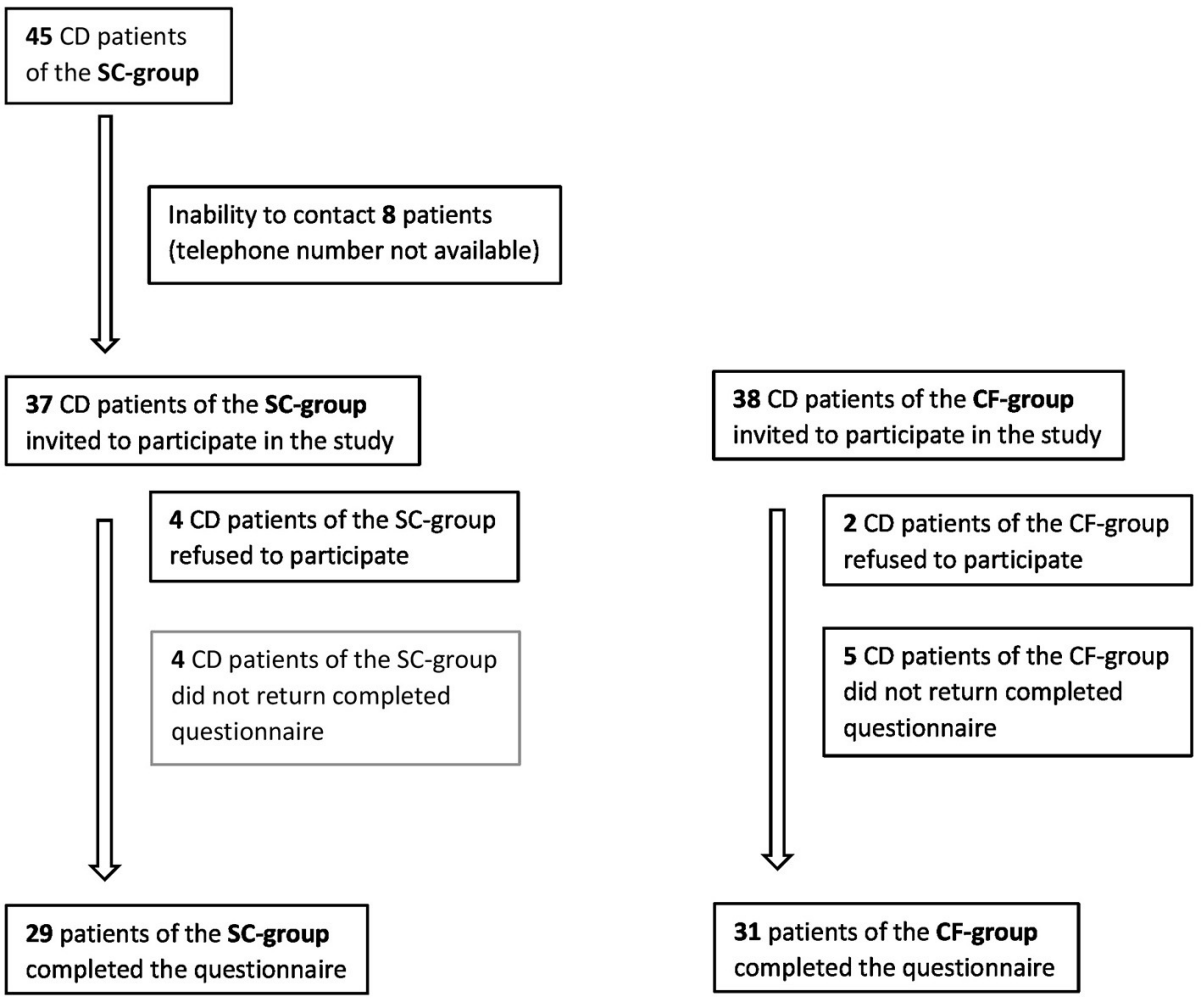
Flow chart of study participants for the screen-detected group and for the case-finding group. SC, screen-detected (group); CF, case-finding (group); CD, celiac disease.

Out of 45 patients belonging to the SC-group, it was possible to contact only 37, because for the remaining eight, an updated telephone number was not available, whereas for the CF-group, 38 patients were invited to participate (Figure 1).

Out of 75 patients, 60 (80%) completed the questionnaire, six (two males + two females for the SC-group and two males for the CF-group) did not consent to the study and were excluded, whereas nine patients (two males + two females for the SC-group and two males + three females for the CF-group) did not return completed questionnaires. Specifically, 29 patients of the SC-group (24 females) and 31 patients of the CF-group (22 females) answered the questionnaire (Table 1 and Figure 1).

**Table 1:** Characteristics of the screen-detected group and of the case-finding group.

	**SC-group (*N* = 29)**	**CF-group (*N* = 31)**	***p*-value**
**Gender (female)**	24 (82.7)	22 (71.0)	0.439
**Age**			
Median [IQR]	18.0 [IQR 16.0–19.0]	17.0 [IQR 15.5–18.0]	0.114
**Age at diagnosis**			
Median [IQR]	7.0 [IQR 6.0–7.0]	9.0 [IQR 5.5–11.5]	0.007^*^
**Follow-up (four-modality analysis)**			
- Referral center for CD (annual follow up)	11 (37.9)	29 (93.5)	<0.001^*^
- Referral center for CD (last checkup >3 years before)	7 (24.1)	0	
- Primary care	6 (20.7)	2 (6.5)	
- None	5 (17.2%)	0	
**Duration of disease**			
Median [IQR]	11.0 [IQR 10.0–12.0]	8.0 [IQR 6.5–11.0]	0.002^*^
**CD-QoL score**			
Median [IQR]	87.0 [IQR 82.0–92.0]	87.0 [IQR 82.0–90.5]	0.711

* *p < 0.05*.

In the SC-group, median age at the time of enrollment was 18.0 years [IQR 16.0–19.0], median age at diagnosis 7.0 years [IQR 6.0–7.0], and median duration of disease (i.e., time from diagnosis) 11.0 years [IQR 10.0–12.0]. In the CF-group, median age at time of enrollment was 17.0 years [IQR 15.5–18.0], median age at diagnosis 9.0 years [IQR 5.5–11.5], and median duration of disease 8.0 years [IQR 6.5–11.0] (Table 1). Overall, the median disease duration of 60 enrolled subjects was 10.0 years [IQR 8.0–12.0].

**Figure 2:**
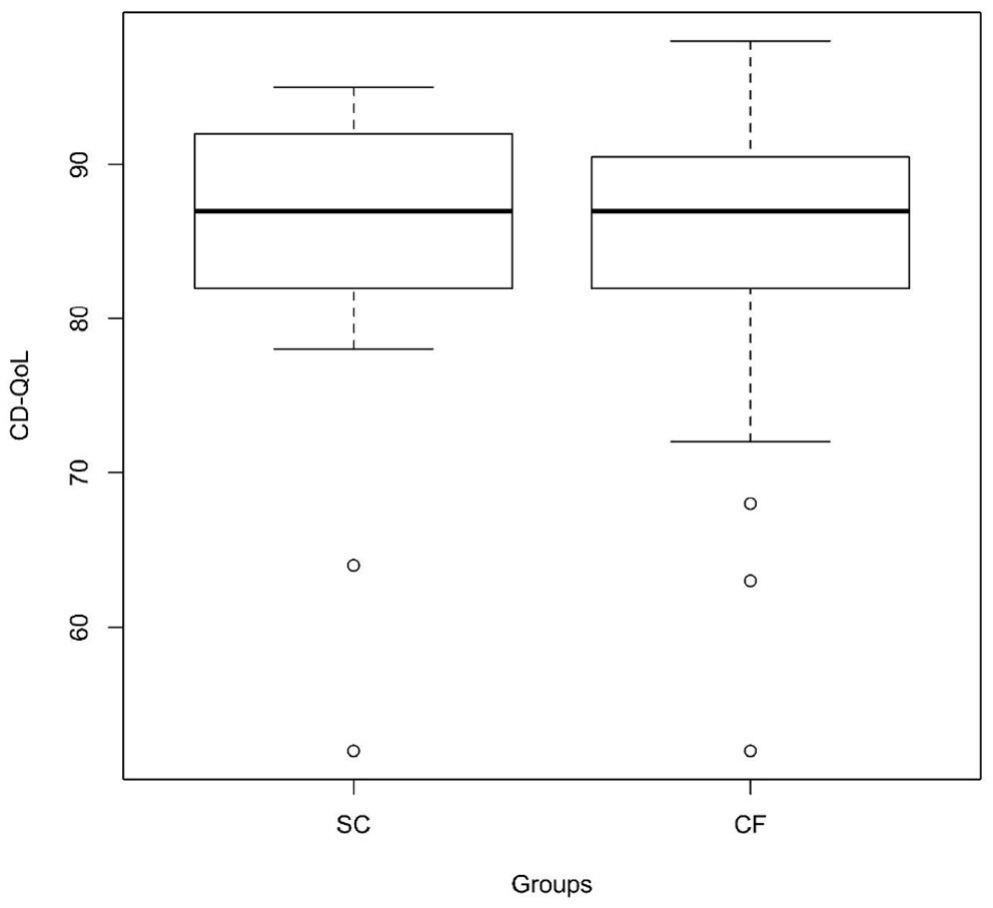
CD-specific QoL in the screen-detected group and in the case-finding group assessed through the Italian version of the CD-QoL scale (*p* = 0.711). SC, screen-detected (group); CF, case-finding (group); CD-QoL, Celiac Disease-Specific Quality of Life.

As regards CD-QoL, both groups scored high on the CD-QoL scale, and no statistically significant differences were observed between the two groups, neither for median total score on the CD-QoL scale (87.0 [IQR 82.0–92.0, range 52–95] in the SC-group and 87.0 [IQR 82.0–90.5, range 52–98] in the CF-group) (*p* = 0.711) (Figure 2 and Table 1) nor for each single question of the scale.

**Figure 3:**
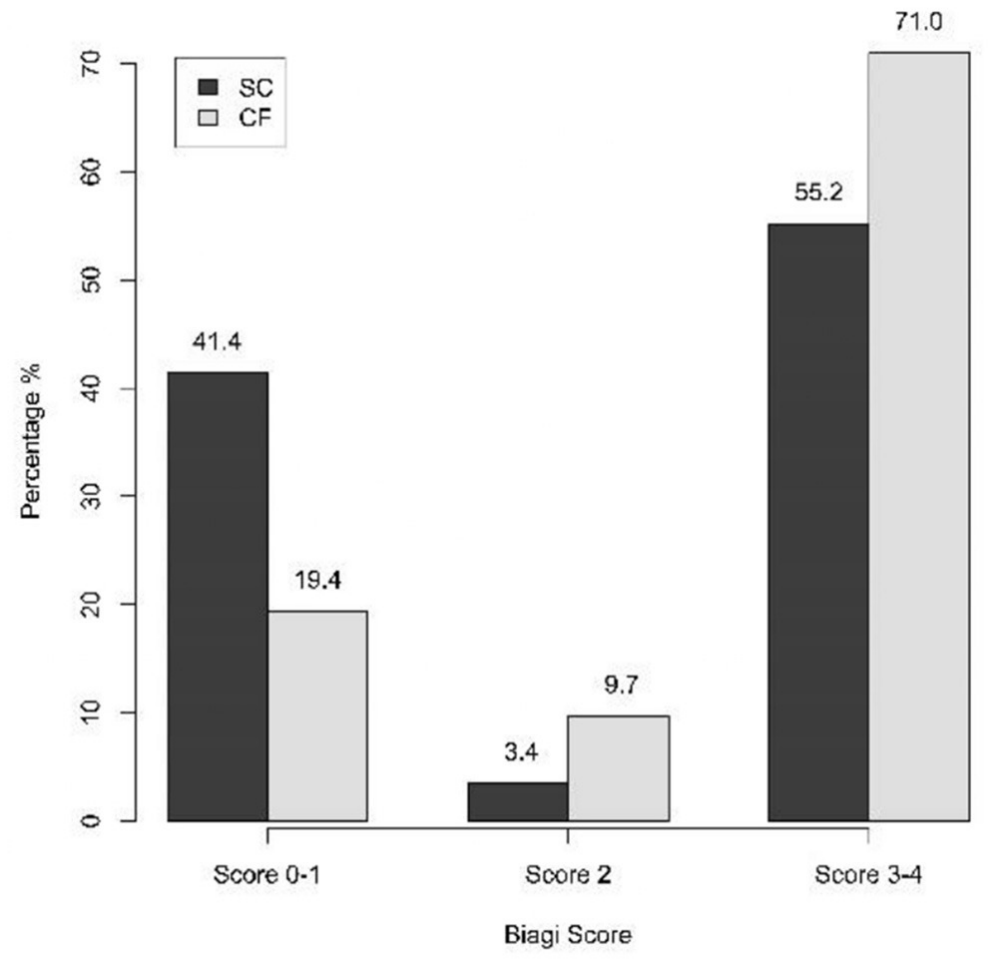
Compliance to the GFD in the SC-group and in the CF-group assessed through the Biagi score (three-category analysis) (*p* = 0.143). SC, screen-detected (group); CF, case-finding (group).

Statistically significant differences were not found in terms of compliance either (*p* = 0.143): 41.4% of the SC-group scored 0-I in the algorithm of Biagi et al. vs 19.4% of the CF-group, while scores of III–IV were achieved by 55.2% of the SC-group and 71.0% of the CF-group (Figure 3). The Biagi score was 0 in 10.3% of the SC-group and in no patient of the CF-group.

**Figure 4:**
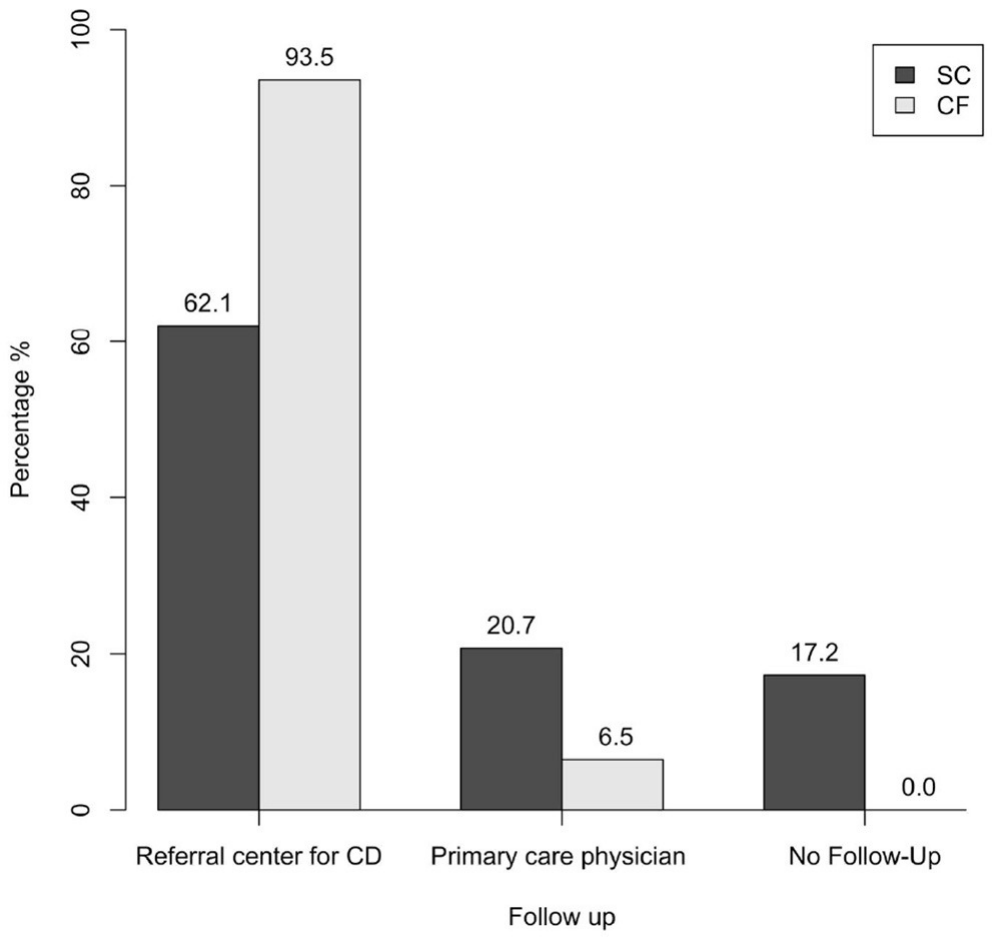
Follow-up of the SC-group and of the CF-group (three-modality follow-up analysis) (*p* < 0.001). SC, screen-detected (group); CF, case-finding (group); CD, celiac disease.

Regarding follow-up for CD, the two groups were compared on the basis of four or three modalities (see Materials and Methods). In both cases, a statistically significant difference was observed when comparing the two groups. Specifically, it was found that 93.5% of patients of the CF-group have been followed up annually at a referral center for CD vs 37.9% of the SC-group (*p* < 0.001) (Table 1); considering also a more dated follow-up at a referral center for CD (three-modality follow-up analysis), the percentage of the SC-group rose to 62.1%, still remaining lower than the percentage observed in the CF-group (*p* < 0.001) (Figure 4).

With regard to follow-up in the primary care, 20.7% of the SC-group have been followed up by their primary care physician, while this was the case in only 6.5% of the CF-group; in addition to this, 17.2% of patients of the SC-group was lost to follow-up, while no patients of the CF-group fell into this category (Figure 4 and Table 1).

**Figure 5:**
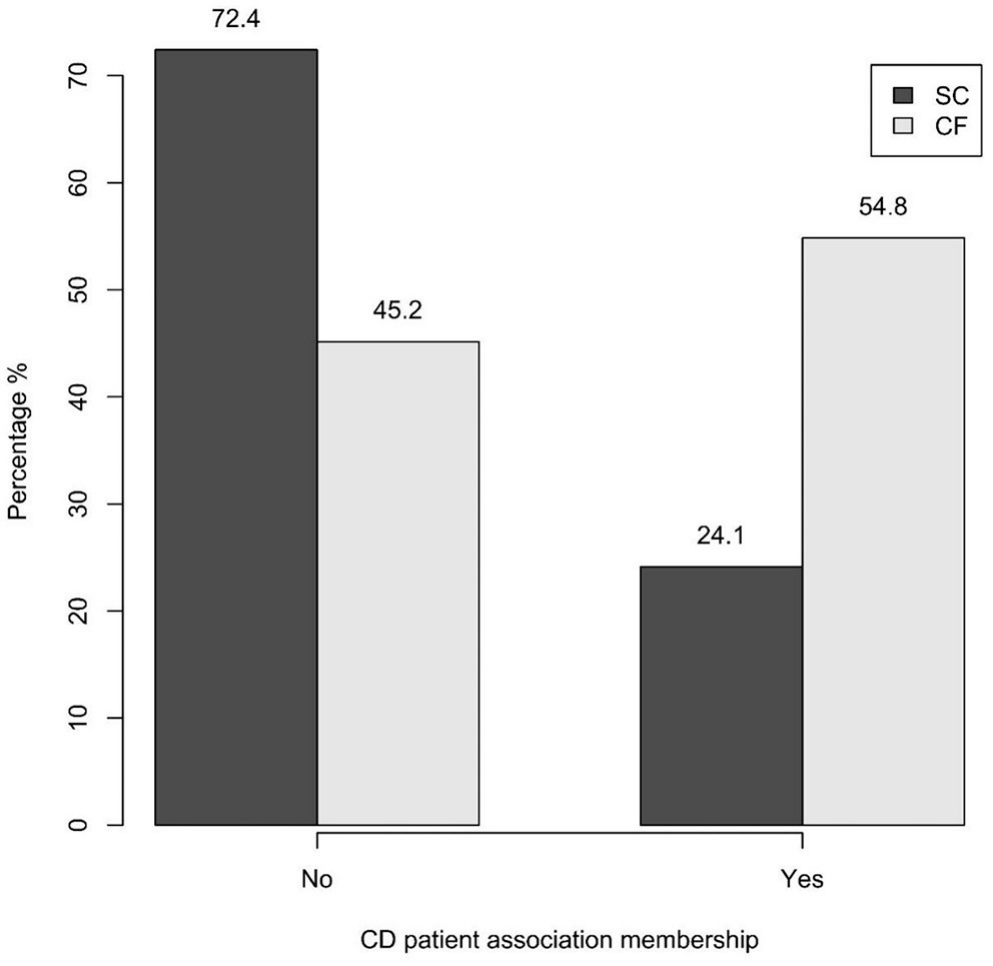
CD patient association membership of the SC-group and of the CF-group (*p* = 0.039). SC, screen-detected (group); CF, case-finding (group); CD, celiac disease.

A statistically significant difference between the two groups was found in relation to CD patient association membership: 24.1% of the SC-group was members of CD associations vs 54.8% of the CF-group (*p* = 0.039) (Figure 5).

**Figure 6:**
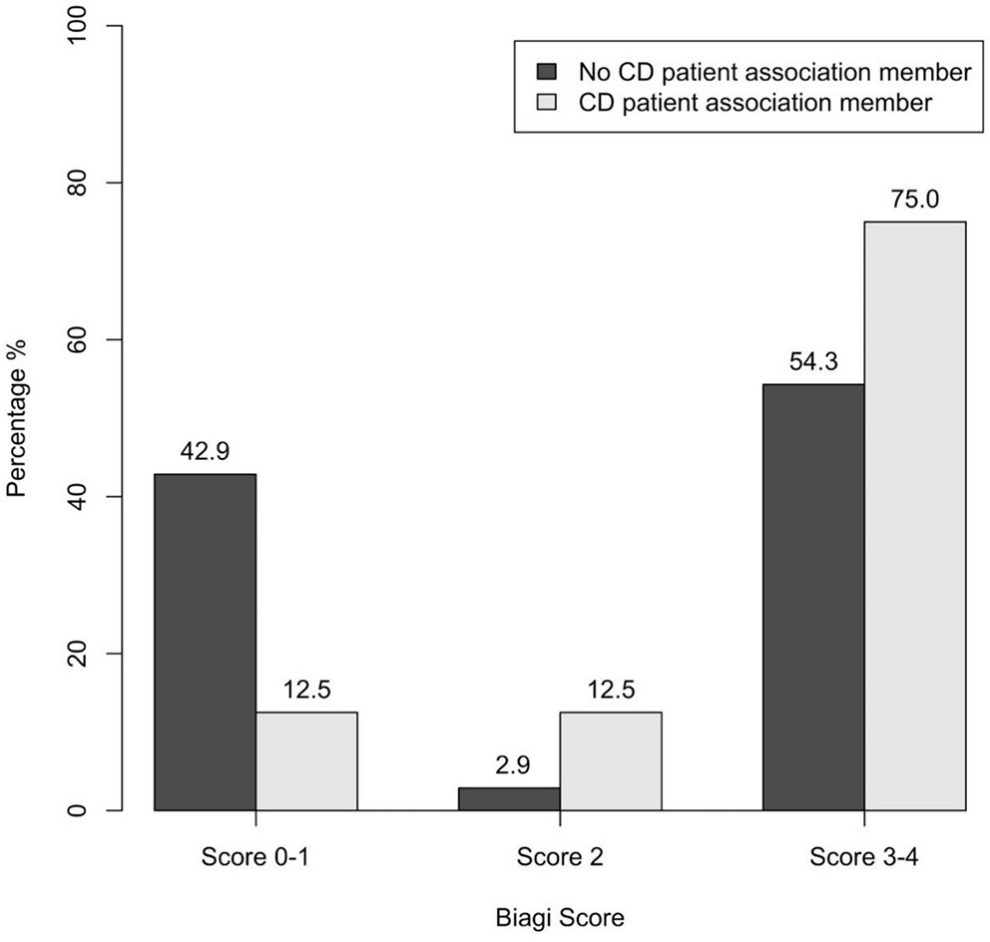
Association between CD patient association membership and dietary compliance assessed through the Biagi score in the 60 patients enrolled (*p* = 0.027). CD, celiac disease.

An association was found between CD patient association membership and compliance to GFD (*p* = 0.027): a Biagi score of 0–I was achieved by 42.9% of non-members vs 12.5% of members (Figure 6). Specifically, the *post-hoc* analysis revealed an association between poor compliance (Biagi score 0–I) and non-membership of the patient and/or of a first-degree family member (*p* = 0.013).

No association was observed between CD association membership and CD-QoL. No association was found between follow-up modalities and CD-QoL or with the Biagi score.

The two groups of the study did not show significant gender differences (*p* = 0.439). A statistically significant difference was found when comparing the two groups' median age at the time of diagnosis (*p* = 0.007) and median duration of disease (*p* = 0.002), but not with respect to median age at the time of enrollment for this study (*p* = 0.114) (Table 1). A statistically significant difference was also found between the group attending follow-up at a referral center and the group of patients who were lost to follow-up whose duration of the disease was longer (*p* = 0.018).

Finally, no association was observed between CD-QoL and compliance in our cohort.

## Discussion

The debate on “mass-screening vs. case-finding” as the best diagnostic strategy for CD is still open, specifically in regard to potential implications for QoL and adherence to the GFD (7, 15–23).

In our study, we found no statistically significant differences in dietary adherence or in CD-QoL between screen-detected and clinically detected CD patients. These findings are in agreement with evidences from short-term follow-up studies that have also shown good adherence to the GFD and a good QoL in screen-detected CD patients (19, 24–27).

A prospective Finnish study from 2017 (24) on a large pediatric CD cohort showed, after a 4-year follow-up, good adherence to the GFD in at-risk groups diagnosed following a screening strategy, even better than compliance of CD patients whose diagnosis had been suggested on clinical grounds. Moreover, these data confirmed those of a previous study by the same group (19), advocating that the diagnostic approach had no impact on adherence to the diet after 1 year from diagnosis.

A 5-year follow-up Swedish study reported a good dietary compliance even in CD adolescents diagnosed following a mass screening (27), and similar results were observed at the 1-year follow-up of the same cohort (25).

Different data came instead from a previous Italian study by Fabiani et al.: only 23% of CD adolescents, diagnosed by means of serological mass screening appeared to maintain strict adherence to the diet after 5 years from diagnosis, compared to 68% of those diagnosed for clinical evidence of disease (28). However, when interpreting the study results by Fabiani et al. it is appropriate to consider not only that they related to a population of adolescents but also that these dated back in 2000: in fact, in the last 20 years, awareness of the disease has progressively increased, and greater availability as well as better palatability of gluten-free products has been achieved. Moreover, it should be highlighted that in Italy there is overall a higher dietary intake of wheat as compared with other countries, and this could lead to greater difficulties for Italian CD patients, who tend to reproduce the pattern of cereal-based product consumption of the general population (29).

Studies on screen-detected adolescents have also shown satisfactory results in terms of QoL (26), and the high scores in the CD-QoL scale found in our study are in line with these data. However, long-term follow-up studies on these topics are scant.

In 2018, Kivelä et al. (30) published a study on adult CD patients diagnosed during childhood, comparing outcomes according to the diagnostic strategy (screening of at-risk subjects vs. case-finding): after an 18-year follow-up, dietary compliance and QoL overlapped in the two groups, and similar evidence was reported from a previous long-term Finnish study (follow-up of 14 years) (31).

The Dutch study by Van Koppen et al. (32) is one of the studies with the longest follow-up (10 years) after a mass screening: in this prospective study on adolescents screened for CD between 2 and 4 years of age, adherence to the diet and QoL showed satisfactory results. However, they have examined patients in an early phase of adolescence, when the subject's diet may still have been under the parents' responsibility, and this may have influenced the excellent results achieved in terms of compliance.

A further objective of our work was the analysis of follow-up practices. Studies suggest that in screen-detected patients, adherence to the diet is favored by adequate follow-up in a well-organized clinical practice (24). A study on pediatric patients and young adults with CD described better adherence to the GFD in patients attending regular follow-up visits at a tertiary care center compared to patients who were lost at specialist follow-up (14). In our study, patients from the SC-group have attended less follow-up consultations at a referral center for CD compared to the CF-group, and the difference between the two groups was statistically significant. Although no association between follow-up modality and compliance has been identified in our study, a decreasing trend in long-term adherence to the diet has been observed in screen-detected patients. Although there are no statistically significant differences between the two groups in this regard, it cannot be excluded that this is attributable to the small sample size.

Finally, poor adherence to the diet was found to be significantly more frequent among non-members of the local CD support group, and this is in line with literature data (33).

To our knowledge, this is one of the studies with the longest follow-up (median 11 years) of a screened CD population and the first one ever performed outside northern Europe.

In light of our results, the diagnostic strategy does not seem to impact QoL and dietary compliance. According to these findings, a screening strategy for the diagnosis of CD could outweigh a case-finding approach. However, some considerations should be kept in mind. Although our results echo previous studies (mainly short-term follow-up studies), long-term data are limited and refer mostly to screened populations composed of adult patients or at-risk groups. Therefore, both the age and membership to an at-risk category could convey a greater patient “responsibility” toward the diet and could have played a role in achieving compliance. This aspect may be different for CD adolescents, even more if they have been identified through a mass screening. Unlike previous works, ours targeted adolescents/young adults: albeit similar results in terms of compliance and QoL in relation to the diagnostic strategy were encountered, adherence to the GFD was found overall less rigorous in the SC-group; in addition, it was lower (55.2%) than the compliance rate observed in other long-term studies (72.9–83.0%) (30–32) considering patients in early adolescence or adulthood.

It is also noteworthy that our SC-group less frequently attended the tertiary care center for follow-up compared to the CF-group, showing a greater tendency to rely on primary care or even to skip any kind of follow-up practice. Despite follow-up of CD patients in primary care having been increasingly encouraged in recent years, our results have become definitely food for thought: providing a well-structured follow-up might be necessary, especially for patients identified by screening, and physicians should pay more caution before addressing this group to the primary care setting.

Besides the long follow-up and the peculiar target of age of patients enrolled, the use of validated and standardized questionnaires to evaluate the CD-related QoL and the dietary compliance are strengths of our study. On the other hand, we are aware of some limitations, such as the lack of laboratory tests to assess compliance (serology and gluten peptides in stool and in urine), although inconstant and minor voluntary or involuntary transgressions may not be detected by these methods (34, 35). In addition, there is a gender unbalance of our cohort, but it reflects the epidemiology scenario of CD rather than a greater tendency of the female gender to attend to questionnaires. In fact, the percentage of males and females who refused to participate in this research was similar; moreover, the compared groups did not show significant gender differences, and therefore, it is unlikely that this could have influenced our results. Other potential limitations may be (1) selection bias because not all the invited subjects answered the questionnaire, (2) the main selection of patients for the CF-group in the context of outpatient visits (which partly weakens our speculations on follow-up practices); and (3) the lack of information about social backgrounds for patients and their families. Finally, the screened population of this study included 45 CD patients diagnosed in a previous work, so a selection bias may occur. In spite of this, the uniqueness of this population with such a long median duration of disease should be recognized.

In conclusion, we found no significant differences in terms of long-term QoL and compliance in young adults as a consequence of a screening policy compared to case-finding.

We hope that further long-term follow-up studies on wider populations could confirm these data and finally fuel a large-scale screening program for CD diagnosis.

## Data Availability Statement

The raw data supporting the conclusions of this article will be made available by the authors, without undue reservation.

## Ethics Statement

The studies involving human participants were reviewed and approved by Ethical Committee of Sapienza University of Rome, Italy. Written informed consent to participate in this study was provided by the participants' legal guardian/next of kin.

## Author Contributions

DI conceived and designed the study, enrolled participants, collected the data, and drafted the manuscript. FV conceived and designed the study, enrolled participants, and drafted the manuscript. AV and AD took care of statistical analyses and interpretation of data and drafted the manuscript. SC supervised the whole project, contributed data interpretation, and critically reviewed the manuscript for important intellectual content. RL contributed data analysis and interpretation and critically revised the manuscript. MM conceived and designed the study, participated in enrolment of patients, coordinated and supervised data collection, and critically reviewed the manuscript. All authors approved the final manuscript as submitted and agree to be accountable for all aspects of the work.

## Funding

This paper has been supported by an unconditional grant from Pharmextracta S.p.A which covered 80% of the publication fee. The funder was not involved in the study design, collection, analysis, interpretation of data, the writing of this article or the decision to submit it for publication.

## Conflict of Interest

The authors declare that the research was conducted in the absence of any commercial or financial relationships that could be construed as a potential conflict of interest.

## Publisher's Note

All claims expressed in this article are solely those of the authors and do not necessarily represent those of their affiliated organizations, or those of the publisher, the editors and the reviewers. Any product that may be evaluated in this article, or claim that may be made by its manufacturer, is not guaranteed or endorsed by the publisher.

## Abbreviations

QoL: Quality of life
CD: Celiac disease
CD-QoL: Celiac Disease-Specific Quality of Life
SC-group: screen-detected group
CF-group: case-finding group
GFD: gluten-free diet.
